# Does hospitalisation improve oral anticoagulant optimisation in patients with atrial fibrillation?

**DOI:** 10.1111/eci.70011

**Published:** 2025-02-14

**Authors:** Belayneh Kefale, Gregory M. Peterson, Corinne Mirkazemi, Nathan B. Dwyer, Mohammed S. Salahudeen, Janette Radford, Camille M. Boland, Woldesellassie M. Bezabhe

**Affiliations:** ^1^ School of Pharmacy and Pharmacology, College of Health and Medicine University of Tasmania Hobart Tasmania Australia; ^2^ Clinical Pharmacy Unit and Research Team, Department of Pharmacy, College of Medicine and Health Sciences Bahir Dar University Bahir Dar Ethiopia; ^3^ Royal Hobart Hospital Hobart Tasmania Australia; ^4^ University of Tasmania Launceston Clinical School Launceston Tasmania Australia

**Keywords:** appropriateness, atrial fibrillation, bleeding, hospitalisation, oral anticoagulant, stroke

## Abstract

**Background:**

Hospitalisation offers an opportunity for medication review and correction, yet it has received little attention. We aimed to evaluate oral anticoagulant (OAC) use in patients with atrial fibrillation at hospital admission and discharge and determine whether hospitalisation improves care.

**Methods:**

We conducted an observational study at the Royal Hobart Hospital, Australia, in patients with atrial fibrillation. The appropriateness of stroke‐prevention therapy at admission and discharge was evaluated using Australian guidelines. Factors associated with correcting inappropriate OAC therapy were identified using multiple logistic regression.

**Results:**

Among 902 patients, 47.1% (*n* = 425) were receiving inappropriate OAC therapy at admission. The most common errors included lack of OAC therapy (58.6%, *n* = 249) and underdosing of direct‐acting OACs (15.5%, *n* = 66). OAC therapy appropriateness at discharge was assessed for 844 patients; 73.8% were receiving appropriate therapy (versus 53.8% at admission (*p* < .001)). Specifically, 49.0% (*n* = 191) of the admission therapy errors were corrected. Correction was more likely in patients admitted to the stroke (adjusted odds ratio [aOR]: 16.93, 95% CI: 1.31–218.48) or cardiology wards (aOR: 4.10, 95% CI: 1.94–8.64), and if bleeding occurred during hospitalisation (aOR: 4.01, 95% CI: 1.07–14.99). Conversely, receiving rivaroxaban at admission (aOR: .23, 95% CI: .11–.51) and having a medium or high bleeding risk (ORBIT score ≥3) (aOR: .46, 95% CI: .25–.84) decreased the likelihood of correction.

**Conclusion:**

Hospitalisation improved OAC therapy appropriateness; however, 51.0% of patients admitted with inappropriate therapy continued without correction. An intervention that enhances the hospital care team correcting inappropriate OAC therapy is warranted.

## Graphical abstract

Hospitalisation increased the appropriateness of OAC therapy from 53.8% at admission to 73.8% at discharge (*p* < 0.001). Despite these improvements, approximately a quarter (26.2%) of patients with AF still received inappropriate OAC therapy at hospital discharge, highlighting a critical gap in optimising OAC therapy.

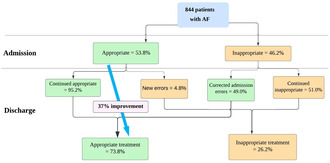

## 1 INTRODUCTION

Atrial fibrillation (AF) is the most common cardiac arrhythmia diagnosed in clinical practice.^1^ AF affects approximately half a million people in Australia.^2^ The prevalence of AF is rapidly increasing, especially as the population ages,^3^ leading to increased morbidity, mortality, and healthcare costs.^4^ It increases the risk of stroke and death five‐ and two‐fold, respectively^5^, ^6^ and 20%–30% of all stroke cases are due to AF.^7^ AF also increases the risk of dementia.^8^ Over the last two decades, Australia's burden of AF‐related hospitalisations increased by 295%.^9^

Stroke is a detrimental consequence of AF, but it is largely preventable with oral anticoagulants (OACs).^7^, ^10^ OACs reduce stroke risk by two‐thirds and all‐cause mortality by one‐fourth.^11^ For decades, warfarin was the only OAC option,^12^ but it has drawbacks, including drug and dietary interactions and the requirement for close monitoring. Direct‐acting oral anticoagulants (DOACs) have revolutionised thromboprophylaxis in AF, offering therapeutic options with better safety and effectiveness.^13^, ^14^ Over the last 10 years, the use of OACs has doubled worldwide, largely as a result of the introduction of DOACs.^15^ In Australia, DOACs accounted for 90% of OAC usage in 2022–23.^16^

However, inappropriate OAC use is still common,^17^, ^18^ causing significant health problems and financial burdens.^18^ The prescribing of OACs for stroke prevention in patients with AF often does not comply with evidence‐based guidelines, resulting in underuse in high‐risk patients and overuse in low‐risk ones.^19^, ^20^, ^21^ Nationwide consecutive analyses using a large Australian general practice dataset revealed that inappropriate OAC use (including not prescribing an OAC for guideline‐eligible patients, prescribing an OAC for guideline‐ineligible patients, and incorrect dosing) remains high among patients with AF.^17^, ^22^, ^23^ Only half (52%) of patients with AF and at high stroke risk were receiving OAC therapy, and nearly a quarter of those receiving DOACs were taking inappropriate doses.^23^ Stroke risk reassessment was also suboptimal: almost one‐third of patients initially at low to moderate risk, later reclassified as high risk over 9 years, did not receive OACs. Among those who received OACs, there was a median delay of 2 years in OAC initiation after becoming at high risk of stroke.^17^

Inappropriate OAC use in patients with AF increases the risk of hospitalisations.^24^ Conversely, hospitalisation for any cause represents an opportunity to identify patients with AF who are not receiving appropriate OAC therapy^25^ and to improve the use of OACs by involving a multidisciplinary team. However, the potential of hospitalisation to improve OAC therapy in patients with AF remains largely unevaluated. Therefore, this study aimed to assess the appropriateness of OAC use in patients with AF upon hospital admission and discharge and to determine whether hospitalisation offers an opportunity to improve care.

## 2 METHODS

### 2.1 Study design, setting and participants

This study was a retrospective observational study at the Royal Hobart Hospital (RHH), a 500‐bed teaching hospital and the main public hospital in Tasmania, Australia. Patients aged 18 years or older with AF admitted for any reason to the RHH between January 1, 2021 and December 31, 2022 were identified from the RHH Admitted Patient Care National Minimum Dataset and the hospital's digital medical records. Patients were excluded if their AF diagnosis was recent (within 3 months prior to their index admission during the study period), their AF was secondary to an acute illness, they had a single episode of AF that resolved by cardioversion without recurrence, they had a hospital stay of less than 24 h, or if data was missing regarding their renal function, weight or OAC drug dosage. Only the first admission during the study period, with complete data, was available to assess the appropriateness of OAC therapy. The University of Tasmania Human Research Ethics Committee (Approval number: 28333) approved this research and waived the consent requirement due to the retrospective observational nature of the study. Approval was also obtained from Tasmanian Department of Health Research Governance (SSA630).

### 2.2 Data collection tool and procedures

The data collection form was developed using peer‐reviewed journal findings and the Australian AF guidelines.^26^, ^27^ We collected admission and discharge dates, reason for admission, admission ward, sociodemographic characteristics, medical history, serum creatinine and estimated glomerular filtration rate (eGFR), platelet count, and haemoglobin level, as well as anticoagulant therapy status at admission and discharge, and rehospitalisation status (within 3 months) and its cause. In‐hospital bleeding (gastrointestinal, intracranial, and bleeding from other sites), thromboembolic events (ischaemic stroke, systemic embolism, transient ischaemic attack, deep vein thrombosis and others) and all‐cause mortality were collected. We calculated the scores for CHA_2_DS_2_‐VA (1 point each for each of congestive heart failure, hypertension, diabetes mellitus, vascular disease, and age 65–74 years, with 2 points for each of age ≥75 years and stroke/transient ischaemic attack) and ORBIT (1 point each for age >74 years, eGFR <60 mL/min/1.73m^2^, treatment with antiplatelet agents, and 2 points each for prior bleeding and low haemoglobin level (<13 g/dL for males or <12 g/dL for females)).^28^, ^29^ We also estimated creatinine clearance (CrCl) using the Cockcroft‐Gault equation.^30^

OAC treatment was evaluated against the Australian AF guidelines.^26^ The guidelines recommend estimating stroke risk using the CHA_2_DS_2_‐VA score (sexless CHA_2_DS_2_‐VASc score). They recommend that in individuals with a CHA_2_DS_2_‐VA score of ≥2 (i.e., considered to be at high risk of stroke), an OAC be used, and in those with a score of 1 (moderate risk), OAC therapy be considered. In this study, patients with a score of 2 or higher (without a contraindication to OAC therapy) were deemed to be receiving appropriate therapy if they were taking an OAC. Patients with a score of 1, who had no contraindication to OAC therapy, were deemed to be on appropriate therapy irrespective of whether they were taking an OAC or not. Conversely, in individuals with a score of 0 (low risk), OAC therapy was deemed inappropriate.^26^

DOAC dosing appropriateness for each patient, based on individual characteristics, such as age, weight, serum creatinine or CrCl, and considering the current Australian guidelines for reduced doses, was defined as follows: (i) dabigatran 150 mg twice daily (normally) or 110 mg twice daily if the patient was aged ≥75 years or had a CrCl of 30–50 mL/min or was at high bleeding risk (combination with dual antiplatelet therapy); (ii) rivaroxaban 20 mg daily (normally) or 15 mg daily if CrCl was 15–49 mL/min or in combination with dual antiplatelet therapy; and (iii) apixaban 5 mg twice daily (normally) or 2.5 mg twice daily if at least two of the following were present: age ≥80 years, weight ≤60 kg or serum creatinine >133 μmol/L.^31^ A CrCl <15 mL/min for rivaroxaban, <25 mL/min for apixaban, and < 30 mL/min for dabigatran was considered a contraindication.^31^ Platelet counts <50 × 10^9^/L and active bleeding were also considered contraindications for OAC therapy.^31^ All DOACs were considered contraindicated in the presence of dialysis, mechanical heart valve or rheumatic mitral stenosis, or hepatic disease with associated coagulopathy, including Child‐Pugh C.^32^, ^33^ Decompensated liver disease or a deranged baseline clotting screen (initial INR >1.5) was considered contraindications for warfarin.^32^, ^33^

Each patient's treatment at admission and discharge was evaluated, considering their individual characteristics and OAC eligibility criteria and classified as either guideline‐concordant anticoagulant therapy (hereafter referred to as appropriate stroke‐prevention therapy) or guideline non‐concordant anticoagulant therapy (hereafter referred to as inappropriate stroke‐prevention therapy). Inappropriate therapy included cases where either no OAC was prescribed despite the patient being deemed to require it (CHA_2_DS_2_‐VA ≥2) or an OAC was prescribed when deemed not suitable (e.g., CHA_2_DS_2_‐VA of 0 or known contraindication to OACs). Inappropriate therapy also included cases when either warfarin was used in the absence of conditions preventing DOAC use, or a DOAC was prescribed at a non‐guideline‐recommended dose.

The appropriateness of OAC therapy upon admission was compared with that at discharge in patients with complete data. Patients were then categorised into four groups: continued appropriate treatment, continued inappropriate treatment, corrected treatment errors, and made new treatment errors.

### 2.3 Data analysis

We used descriptive statistics to summarise patient characteristics. Categorical variables were presented as frequencies and percentages (%) and compared using *χ* ^2^ tests. Continuous variables were presented as means ± standard deviations (SD) or medians with interquartile ranges (IQR). For normally distributed data, comparisons were made using the student's t‐test. For non‐normally distributed variables, we used the Mann–Whitney *U* test. Additionally, the McNemar test was used to determine whether there was a significant change in OAC therapy appropriateness from hospital admission to discharge.

A logistic regression model was performed (after checking for multicollinearity using the Variance Inflation Factor, with values <5 indicating low correlation) to identify factors associated with correcting OAC therapy errors during hospitalisation. Initially, we employed univariate logistic regression to assess the impact of individual factors on correcting these errors. This analysis included all collected variables, as outlined under the subheading ‘Data Collection Tool and Procedures.’ Variables with a *p*‐value <.2 from this analysis were subsequently included in a multivariate logistic regression model to identify factors independently related to the correction of admission therapy errors. Multivariate logistic regression was also used to identify factors associated with appropriate OAC use at hospital admission and discharge. A two‐sided *p*‐value <.05 was considered statistically significant. All statistical analyses were performed using IBM SPSS Statistics software, version 29.0 (IBM Corp., Armonk, NY, USA).

## 3 RESULTS

### 3.1 Study patient characteristics

**FIGURE 1:**
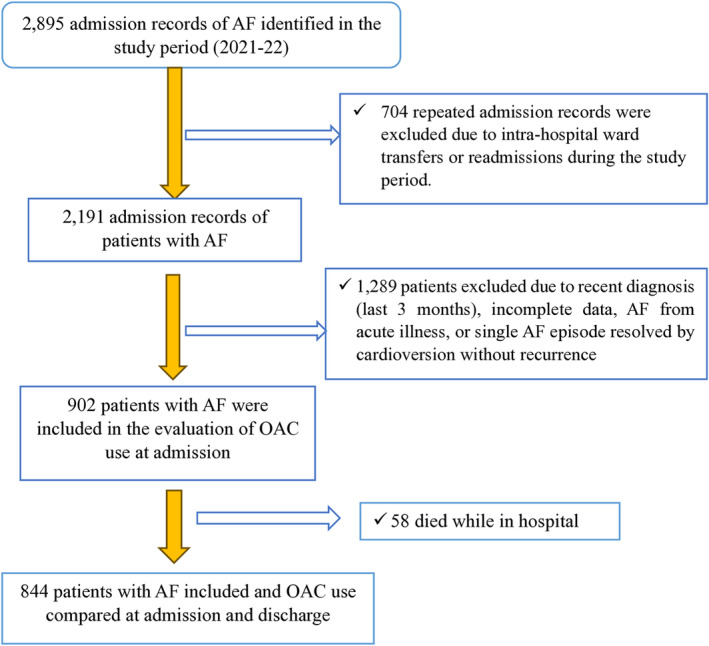
Study participant selection flowchart.

From January 1, 2021, to December 31, 2022, a total of 2895 patients with AF were identified. Of these, 902 patients met the inclusion criteria (Figure 1). The mean age was 76.8 years (SD 11.0); 59.8% were male. Thirty‐nine percent of patients were admitted to general medical wards, 20.2% to surgical wards, and 19.3% to cardiology wards (Table 1).

**TABLE 1:** Socio‐demographic and clinical characteristics of participants, stratified by OAC therapy appropriateness at hospital admission.

Variables		Overall [*n* = 902, 100%]	Appropriate therapy [*n* = 477, 52.9%]	Inappropriate therapy [*n* = 425, 47.1%]	Between group *p*‐value
Gender, *n* (%)	Male	539 (59.8)	279 (51.8)	260 (48.2)	.412
	Female	363 (40.2)	198 (55.5)	165 (45.5)	
Age (yrs.), mean [SD]		76.8 (11.0)	75.7 (11.1)	77.9 (10.8)	.004
Weight (kg), median (IQR)		80 [68–93]	80 [69–95]	78 [65.8–90]	.159
Cause of admission	Stroke	85 (9.4)	32 (37.6)	53 (62.4)	< .001
	Atrial fibrillation	115 (12.7)	79 (68.7)	36 (31.3)	
	Surgery unrelated to stroke	189 (21.0)	85 (45.0)	104 (55.0)	
	Other medical admissions	513 (56.9)	281 (54.8)	232 (45.2)	
Admission ward	Stroke	92 (10.2)	36 (39.1)	56 (60.9)	< .001
	Cardiology	174 (19.3)	109 (62.6)	65 (37.4)	
	General medicine	352 (39.0)	199 (56.5)	153 (43.5)	
	Surgical	182 (20.2)	81 (44.5)	101 (55.5)	
	Geriatric	36 (4.0)	17 (47.2)	19 (52.8)	
	Emergency	21 (2.3)	15 (71.4)	6 (28.6)	
	Oncology	21 (2.3)	9 (42.9)	12 (57.1)	
	Others	24 (2.7)	11 (45.8)	13 (54.2)	
Length of stay (d), median (IQR)		7 [3–13]	6 [3–11.5]	7 [3–14]	.022
CHA_2_DS_2_VA‐score, mean (SD)		3.8 (1.6)	3.6 (1.7)	4.0 (1.5)	<.001
CHA_2_DS_2_VA‐score ‐absolute value, n (%)	0	13 (1.4)	8 (61.5)	5 (38.5)	<.001
	1	47 (5.2)	45 (95.7)	2 (4.3)	
	2	150 (16.6)	72 (48.0)	78 (52.0)	
	3	163 (18.1)	99 (60.7)	64 (39.3)	
	4	233 (25.8)	109 (46.8)	124 (53.2)	
	5	152 (16.9)	79 (52.0)	73 (48.0)	
	6	98 (10.8)	41 (41.8)	57 (58.2)	
	7	36 (4.0)	18 (50.0)	18 (50.0)	
	8	10 (1.1)	6 (60.0)	4 (40.0)	
ORBIT score, mean (SD)		2.5 (2.9)	2.4 (3.7)	2.6 (1.5)	.269
ORBIT score ‐categorical, *n* (%)	0–2/low	488 (54.1)	293 (60.0)	195 (40.0)	<.001
	3/medium	183 (20.3)	79 (42.2)	104 (56.8)	
	≥4/high	231 (25.6)	105 (45.5)	126 (54.5)	
CHF, *n* (%)		326 (36.1)	184 (56.4)	142 (43.6)	.107
Hypertension, *n* (%)		566 (62.7)	277 (48.9)	289 (51.1)	.002
Diabetes, *n* (%)		239 (26.5)	116 (48.5)	123 (51.5)	.116
Stroke, *n* (%)		190 (21.1)	95 (50.0)	95 (50.0)	.370
Vascular disease, *n* (%)		558 (61.9)	276 (49.5)	282 (50.5)	.009
Bleeding history, *n* (%)		65 (7.2)	31 (47.7)	34 (52.3)	.384
CKD, *n* (%)		152 (16.9)	76 (50.7)	76 (49.3)	.435
Cancer, *n* (%)		97 (10.8)	52 (53.6)	45 (43.4)	.915
Dementia, *n* (%)		164 (18.2)	84 (52.5)	80 (48.8)	.666
Admission haemoglobin, (g/L), mean (SD)		127.6 (23)	129.5 (22.7)	125.4 (23.2)	.008
Admission platelets (×10^9^/L), median [IQR]		226 [173.7–286.9]	228 [176–288]	221.5 [172–284]	.418
Admission eGFR (mL/min) /1.73 m^2^, mean (SD)		62.4 (23.3)	62.5 (23.1)	62.3 (23.7)	.621
Admission creatinine (μmol/L), median [IQR]		91.0 [74–119]	93 [74–119]	90 [73–118]	.455

Abbreviations: CHF, congestive heart failure; CKD, chronic kidney disease; eGFR, estimated glomerular filtration rate; IQR, interquartile range; SD, standard deviation.

### 3.2 Appropriateness of stroke‐prevention therapy

Of 902 patients with AF, 52.9% (*n* = 477) were deemed to be receiving appropriate stroke‐prevention therapy on admission. Table 1 shows study participants' baseline sociodemographic and clinical characteristics according to their stroke‐prevention therapy appropriateness status. At admission, multivariate logistic regression analysis determined that only having a medium or high bleeding risk (as determined by their ORBIT score) was associated with receiving inappropriate stroke‐prevention therapy (Table S1). At hospital discharge, patients who had been initially admitted to specialised cardiology (aOR: 1.9, 95% CI: 1.1–3.3, *p* = .019) or stroke (aOR: 13.8, 95% CI: 1.5–127.6, *p* = .021) wards were significantly more likely to receive appropriate therapy. Additionally, admission due to AF (aOR: 2.1, 95% CI: 1.1–3.9, *p* = .030) and the presence of heart failure as a comorbidity (aOR: 1.6, 95% CI: 1.1–2.4, *p* = .026) were associated with increased likelihood of receiving appropriate stroke‐prevention therapy at discharge (Table S1). Conversely, advancing age (aOR: .97 per year, 95% CI: .96–.99, *p* < .001) was associated with lower odds of receiving appropriate stroke‐prevention therapy (Table S1).

Among the patients deemed to be receiving inappropriate stroke‐prevention therapy on admission to the hospital, the most common errors were lack of OAC therapy despite being deemed by guidelines to require it (58.6%, 249/425) and DOAC underdosing (15.5%, 66/425) (Table 2).

**TABLE 2:** Stroke prevention therapy appropriateness by OAC and the types of errors at admission.

Patient variables		Overall [*n* = 902, 100%]	Appropriate therapy [*n* = 477, 52.9%]	Inappropriate therapy [*n* = 425, 47.1%]
Admission anticoagulant therapy, *n* (%)	Nil OAC	290 (32.2)	41 (14.1)	249 (85.9)
	Apixaban	276 (30.6)	209 (75.7)	67 (24.3
	Rivaroxaban	228 (25.3)	160 (70.2)	68 (29.8)
	Dabigatran	46 (5.1)	39 (84.8)	7 (15.2)
	Warfarin	62 (6.9)	28 (45.2)	34 (54.8)
Contraindication to DOAC, *n* (%)		153 (16.9)	55 (35.9)	98 (64.1)
Contraindication to OAC, *n* (%)		38 (4.2)	19 (50.0)	19 (50.0)
Specific type of error at admission, *n* (%)	Lacking OAC (underuse)	249 (27.6)	–	249 (58.6)
	DOAC underdosing	66 (7.3)	–	66 (15.5)
	Receiving DOAC despite CI to DOAC	39 (4.3)	–	39 (9.2)
	Receiving warfarin despite no CI to DOAC	32 (3.5)	–	32 (7.5)
	DOAC overdosing	20 (2.2)	–	20 (4.7)
	Receiving OAC despite CI	16 (1.8)	–	16 (3.8)
	Receiving DOAC when deemed not suitable	3 (0.3)	–	3 (0.7)

Abbreviations: CI, contraindication; DOAC, direct oral anticoagulant; OAC, oral anticoagulant.

### 3.3 Change of anticoagulant therapy appropriateness from admission to discharge

**FIGURE 2:**
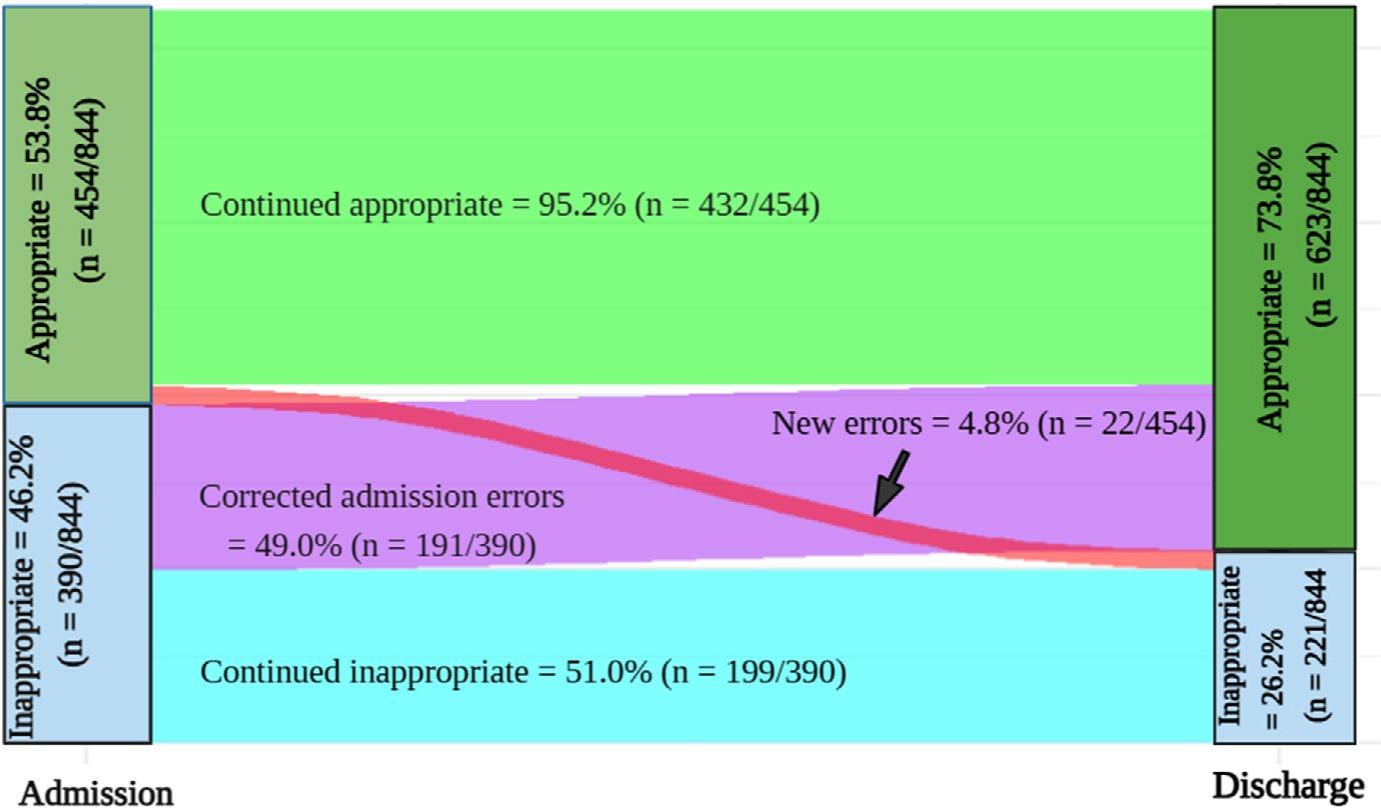
Sankey diagram showing the treatment appropriateness at discharge compared to admission (*N* = 844).

The appropriateness of stroke‐prevention treatment at discharge was assessed for the 844 patients with complete data. Figure 2 shows the changes in treatment appropriateness from admission to discharge. At admission, 53.8% (454/844) of these patients were deemed to be receiving appropriate treatment. This increased to 73.8% (623/844) at discharge (*p* < .001). This led to a 37.2% ([623–454]/454) improvement in OAC appropriateness. Nearly half (49.0%, 191/390) of the patients who were judged to be receiving inappropriate stroke‐prevention treatment at admission were discharged with guideline‐concordant treatment (Table 3 and Figure 2). In comparison, 4.8% (22/454) of those initially receiving appropriate treatment did not continue it at discharge (Figure 2). More than half of the individuals who were deemed to require OAC therapy, but were not receiving it at admission, were discharged with an OAC (56.9%, *n* = 132) (Table 3).

**TABLE 3:** Change of anticoagulant therapy appropriateness from admission to discharge by OAC and error types.

Patient variables		Overall [*n* = 844, 100%]	Continued appropriate treatment *n* = 432, 51.2%	Continued inappropriate treatment *n* = 199, 23.6%	Corrected admission error *n* = 191, 22.6%	Created new error on discharge *n* = 22, 2.6%
Admission anticoagulant therapy, *n* (%)	Nil OAC	271 (32.1)	37 (13.7)	100 (36.9)	132 (48.7)	2 (0.7)
	Apixaban	256 (30.3)	186 (72.7)	31 (12.1)	26 (10.2)	13 (5.1)
	Rivaroxaban	215 (25.5)	146 (67.9)	47 (21.9)	16 (7.4)	6 (2.8)
	Dabigatran	45 (5.3)	39 (86.7)	5 (11.1)	1 (2.2)	0 (0.0)
	Warfarin	57 (6.8)	24 (42.1)	16 (28.1)	16 (28.1)	1 (1.8)
Specific type of error at admission, *n* (%)	Underuse (not receiving OAC despite being deemed to need it)	232 (27.5)	–	100 (43.1)	132 (56.9)	–
	Receiving OAC despite CI	15 (1.8)	–	2 (13.3)	13 (86.7)	–
	Receiving DOAC despite CI to DOAC	33 (3.9)	–	22 (66.7)	11 (33.3)	–
	DOAC under dosing	60 (7.1)	–	45 (75.0)	15 (25.0)	–
	DOAC overdosing	19 (2.3)	–	11 (57.9)	8 (42.1)	–
	Receiving DOAC while guideline‐ineligible (low stroke risk)	3 (0.4)	–	3 (100.0)	0 (0.0)	–
Specific type of error at discharge, *n* (%)	Receiving OAC despite CI	4 (0.5)	–	4 (100.0)	–	0 (0.0)
	Receiving DOAC despite CI to DOAC	21 (2.5)	–	19 (90.5)	–	2 (9.5)
	DOAC under dosing	59 (7.0)	–	51 (86.4)	–	8 (13.6)
	DOAC overdosing	12 (1.4)	–	11 (91.7)	–	1 (8.3)
	Receiving DOAC despite deemed unsuitable	4 (0.5)	–	3 (75.0)	–	1 (25.0)

An in‐depth analysis of inappropriate stroke‐prevention therapy revealed that certain errors often persisted from admission to discharge. These included DOAC underdosing (persisting in 70.0% of patients, *n* = 42/60), DOAC overdosing (47.4%, *n* = 9/19), warfarin use despite no contraindication to a DOAC (43.3%, *n* = 13/30), anticoagulant use despite contraindications (40.9%, *n* = 18/44), and lack of anticoagulation when indicated (37.1%, *n* = 86/232) (Table S2). Table S3 shows the changes in stroke‐prevention therapy appropriateness at discharge for patients who were initially classified as receiving inappropriate therapy at admission.

### 3.4 Factors associated with correction of admission stroke‐prevention therapy errors

In the multivariate analysis, factors increasing the likelihood of correcting an admission stroke‐prevention therapy error were being admitted to a stroke ward (adjusted odds ratio [aOR]: 16.93, 95% CI: 1.31–218.48, *p* = .030), being admitted to a cardiology ward (aOR: 4.10, 95% CI: 1.94–8.64, *p* < .001), and experiencing bleeding during hospital care (aOR: 4.01, 95% CI: 1.07–14.99, *p* = .039). Conversely, receiving rivaroxaban at admission (aOR: .23, 95% CI: .11–.51, *p* < .001) and having medium or high bleeding risk (ORBIT score ≥3) (aOR: .46, 95% CI: .25–.84, *p* = .011) were significantly associated with a decreased likelihood of correction (Table 4).

**TABLE 4:** Univariate and multivariate logistic regression analysis of factors affecting the correction of admission OAC therapy errors.^b^

Variables		Univariate		Multivariate	
		COR (95% CI)	*p*‐Value	AOR (95% CI)	*p*‐Value
Male gender		**1.7 [1.14–2.60]**	.**010**	1.41 [.84–2.35]	.195
Admission ward	General Medicine	1		1	
	Cardiology	**4.5 [2.4–8.7]**	**<.001**	**4.10 [1.94–8.64]**	**<.001**
	Surgery	1.5 [.9–2.6]	.117	6.07 [.85–43.33]	.072
	Stroke	**8.5 [3.8–18.9]**	**<.001**	**16.93 [1.31–218.48]**	.**030**
	Geriatric	.7 [.2–2.1]	.516	.60 [.16–2.23]	.443
	Emergency	.9 [.2–5.1]	.911	.71 [.06–8.24]	.781
	Oncology	.4 [.1–1.9]	.257	.56 [.09–3.44]	.527
	Others	.8 [.2–3.1]	.723	1.02 [.19–5.56]	.986
Reasons for admission	Other medical conditions	1		1	
	Stroke	**4.6 [2.2–9.5]**	**<.001**	.27 [.02–3.27]	.304
	AF	**2.1 [1.0–4.3]**	.**044**	1.49 [.61–3.68]	.341
	General surgery	1.0 [.6–1.7]	.933	.26 [.04–1.79]	.172
Age (years)		.**6 [.4–.9]**	.**007**	.56 [.33–1.02]	.061
History of stroke (Yes)		**2.1 [1.3–3.4]**	.**004**	1.63 [.82–3.26]	.167
History of bleeding (Yes)		2.2 [.96–5.0]	.062	1.15 [.37–3.55]	.812
ORBIT‐score	Low	1		1	
	Medium and high	.6 [.4–.8]	.004	.**46 [.25–.84]**	.**011**
Low haemoglobin level at admission^a^		.**5 [.4–.8]**	.**002**	.93 [.52–1.70]	.323
Bleeding outcome during hospitalisation		**2.9 [1.3–6.4]**	.**009**	**4.01 [1.07–14.99]**	.**039**
OAC at admission	Apixaban	.6 [.4–1.1]	.127	.59 [.28–1.24]	.165
	Rivaroxaban	.**3 [.1–.5]**	**<.001**	.**23 [.11–.51]**	**<.001**
	Dabigatran	.2 [.02–1.3]	.087	.16 [.01–1.96]	.151
	Warfarin	.8 [.4–1.6]	.462	.45 [.17–1.15]	.093
	No treatment	1		1	
Type of inappropriate treatment	Wrong drug or dosage	1		1	
	Lack of treatment	**2.2 [1.5–3. 4]**	**<.001**	2.21 [.85–5.81]	.106
>5 chronic medications		.**7 [.4–1.0]**	.**061**	1.02 [.60–1.71]	.098
Contra indication to DOAC		.7 [.4–1.1]	.101	.46 [.24–1.03]	.057
Contra indication to OAC		**3.3 [1.0–10.3]**	.**043**	5.42 [.74–39.55]	.096

^a^ (<120 g/L, for female and <130 g/L, for male).

^b^ Including only variables eligible for multivariate analysis.

Highlight predictors that are statistically significantly associated with the outcome variables.

## 4 DISCUSSION

OAC therapy is the cornerstone of stroke prevention in AF. Despite its clear benefits, previous studies have shown that OACs are often underused or inappropriately prescribed in patients with AF.^22^, ^25^ This study aimed to quantify the extent of OAC therapy appropriateness at both hospital admission and discharge, and evaluate whether hospitalisation presents an opportunity to optimise OAC therapy.

Our observational evaluation found that overall OAC therapy appropriateness at admission was 52.9%. Patients with AF receiving appropriate therapy at admission had lower bleeding risk scores. Encouragingly, our study revealed that the proportion of patients receiving appropriate OAC therapy increased from 53.8% at admission to 73.8% at discharge, reflecting a 37.2% improvement. Almost half (49.0%) of those with inappropriate treatment at admission were discharged with appropriate treatment. These results highlight the effectiveness of usual multidisciplinary hospital care in improving the appropriateness of OAC therapy. Consistent with a previous study from Israel, which showed a 29.3% improvement in appropriate OAC therapy from admission (34.4%) to discharge (44.5%), hospitalisation, regardless of the cause, can be an effective intervention point for improving OAC therapy and potentially addressing other medication errors as well.^25^

Despite the observed improvement, over a quarter (26.2%) of patients with AF still received inappropriate OAC therapy at hospital discharge, highlighting a critical gap in optimising OAC therapy. The most common errors that were not corrected were a lack of OAC therapy in guideline‐eligible patients and DOAC underdosing. When OAC therapy was not prescribed, stroke risk reassessment was unlikely to be documented, despite previous studies highlighting the dynamic nature of stroke and bleeding risks over time in patients with AF.^17^, ^34^, ^35^ The errors reflect the challenges that practitioners face in balancing the benefits of stroke prevention against the risk of bleeding, which is the most commonly cited concern when prescribing anticoagulants for patients with AF.^17^, ^36^ In line with our previous nationwide analysis,^23^ where 86.4% of patients on inappropriate OAC doses were underdosed, this study also found that most errors resulted from dose reductions not endorsed by guidelines. This may be due to the belief that lower doses reduce bleeding risk. Previous studies similarly reported frequent DOAC underdosing, potentially compromising stroke prevention.^23^, ^27^, ^37^ Systematic reviews further confirm that unwarranted DOAC dose reductions provide no safety benefits (e.g., reduced bleeding) and increase the risk of mortality^38^ and thrombotic events (particularly with apixaban, where underdosing was associated with nearly a 5‐fold increase in the risk of stroke).^24^

Admission to a cardiology (17 times) or a stroke (4 times) ward was associated with a higher likelihood of correcting admission OAC therapy errors compared to a general medical ward, potentially reflecting a higher level of attention on AF and OAC therapy in these specialised settings. Additionally, experiencing bleeding during hospital care increased the likelihood of correcting OAC therapy errors by fourfold. A well‐known complication of anticoagulant therapy and flagged by the Australian Commission on Safety and Quality in Health Care, bleeding is likely to trigger prompt intervention to reduce medication‐related harm.^39^

Conversely, a higher bleeding risk and receiving rivaroxaban at admission were associated with a decreased likelihood of error correction. Although guidelines suggest that a high bleeding risk should not preclude OAC use^40^ and do not support withdrawing or avoiding anticoagulants based on the bleeding risk score,^41^ clinicians may still hesitate to prescribe or adjust therapy for high‐risk patients. Also, clinicians' decisions on OAC therapy may be influenced by patients' concerns about bleeding side effects.^42^

This study provided detailed insights into the appropriateness of OAC therapy at admission and discharge and highlights the effectiveness of usual hospital care in optimising OAC therapy. However, there are some limitations to consider. The retrospective design means some clinical information, such as a history of bleeding, was possibly missing. We did not reassess the CHA_2_DS_2_‐VA score at discharge for the patients who may have been diagnosed with new comorbidities during hospitalisation. Socioeconomic (e.g., educational and economic status) and clinician‐related factors (e.g., experience), which could significantly impact the appropriateness of OAC therapy, were not assessed. Furthermore, patient frailty status, which may significantly influence the use of OAC therapy, was not assessed. Including patients from a single hospital and evaluating OAC therapy appropriateness based on an Australian national guideline also limits the generalisability of the findings. Lastly, we did not assess long‐term clinical outcomes.

## 5 CONCLUSION

Hospitalisation significantly improved OAC therapy appropriateness, yet half of the patients with inappropriate therapy at admission continued to have it at discharge. Uncorrected underdosing and lack of OAC therapy suggest that the clinicians were cautious with respect to avoiding the risk of bleeding. Exploring the barriers and enablers to optimise stroke‐prevention therapy, followed by implementing targeted interventions, appears warranted.

## AUTHOR CONTRIBUTIONS

BK: Methodology, investigation, statistical analysis and writing the original manuscript draft. WMB, GMP and CM: Conceptualisation, methodology, supervision, writing, review and editing of the manuscript. NBD, MSS, JR and CMB: writing, review and editing. All authors read and approved the final version of this manuscript.

## FUNDING INFORMATION

This work was supported by the Royal Hobart Hospital Research Foundation (RHHRF) Incubator Grant 2023. The funders had no role in the design or analysis of the study or the interpretation of the results.

## CONFLICT OF INTEREST STATEMENT

None declared.

## Supporting information


Table S1.

## Data Availability

All data are available in the manuscript or the supplemental files.
