# Supplementary material for: Does hospitalisation improve oral anticoagulant optimisation in patients with atrial fibrillation?
Source: Eur J Clin Invest. 2025 Feb 14;55(4):e70011. doi: 10.1111/eci.70011 (PMC11891822; doi:10.1111/eci.70011)
Supplement: Supplementary file 1 — Table S1. [file ECI-55-e70011-s001.docx]

**Supplementary tables**

Table S1: Univariate and multivariate logistic regression analyses examining the association between patient socio-demographic and clinical characteristics and the appropriateness of stroke-prevention therapy at admission and discharge*.

| **Variables** | | **Admission** | | | | **Discharge** | | | |
| --- | --- | --- | --- | --- | --- | --- | --- | --- | --- |
|  |  | **Univariate** | | **Multivariate** | | **Univariate** | | **Multivariate** | |
|  |  | COR (95% CI) | P-value | AOR (95% CI) | P-value | COR (95% CI) | P-value | AOR (95% CI) | P-value |
| Age (years) | | **0.98 [0.97-0.99]** | **0.004** | 0.98 [0.97-1.00] | 0.076 | **0.96 [0.95-0.98]** | **<0.001** | **0.97 [0.96-0.99]** | **<0.001** |
| Sex (male) | | 0.9 [0.7-1.2] | 0.412 | - | - | **1.5 [1.1-2.0]** | **0.017** | 1.4 [0.96-1.90] | 0.104 |
| Admission ward | General Medicine | 1 |  | 1 |  | 1 |  | 1 |  |
|  | Cardiology | 1.3 [0.9-1.9] | 0.181 | 0.9 [0.6-1.4] | 0.593 | 3.0 [1.8-5.0] | <0.001 | **1.9 [1.1-3.3]** | **0.019** |
|  | Surgery | **0.6 [0.4-0.9]** | **0.009** | 0.7 [0.2-1.9] | 0.453 | 1.0 [0.7-1.5] | 0.960 | 1.9 [0.6-5.7] | 0.269 |
|  | Stroke | **0.5 [0.3-0.8]** | **0.003** | 0.8 [0.3-2.6] | 0.725 | 2.7 [1.4-5.3] | 0.002 | **13.8 [1.5-127.6]** | **0.021** |
|  | Geriatric | 0.7 [0.3-1.4] | 0.286 | 0.7 [0.4-1.4] | 0.342 | 0.6 [0.3-1.3] | 0.221 | 0.8 [0.4-1.8] | 0.584 |
|  | Emergency | 1.9 [0.7-5.1] | 0.187 | 1.5 [0.5-4.0] | 0.471 | 1.9 [0.6-5.8] | 0.262 | 1.4 [0.4-4.6] | 0.551 |
|  | Oncology | 0.6 [0.2-1.4] | 0.225 | 0.5 [0.2-1.4] | 0.188 | 0.5 [0.2-1.3] | 0.139 | 0.4 [0.2-1.1] | 0.090 |
|  | Others | 0.7 [0.3-1.5] | 0.310 | 0.7 [0.3-1.8] | 0.484 | 0.7 [0.3-1.8] | 0.488 | 0.8 [0.3-2.1] | 0.645 |
| Reasons for admission | Other medical conditions | 1 |  | 1 |  | 1 |  | 1 |  |
|  | Stroke | **0.5 [0.3-0.8]** | **0.004** | 0.6 [0.2-1.8] | 0.336 | 1.9 [1.0-3.5] | 0.039 | 0.18 [0.02-1.6] | 0.119 |
|  | AF | **1.8 [1.2-2.8]** | **0.007** | 1.4 [0.9-2.3] | 0.141 | 2.9 [1.6-5.3] | <0.001 | **2.1 [1.1-3.9]** | **0.030** |
|  | General surgery | **0.7 [0.5-0.9]** | **0.021** | 0.9 [0.3-2.4] | 0.841 | 0.8 [0.6-1.2] | 0.360 | 0.5 [0.16-1.4] | 0.178 |
| CHF (yes) | | 1.3 [0.9-1.7] | 0.107 | 1.2 [0.9-1.7] | 0.253 | 1.3 [0.96-1.85] | 0.085 | **1.6 [1.1-2.4]** | **0.026** |
| Hypertension (yes) | | **0.7 [0.5-0.9]** | **0.002** | 0.7 [0.5-1.0] | 0.084 | 0.8 [0.6-1.1] | 0.209 | - | - |
| Diabetes (yes) | | 0.8 [0.6-1.1] | 0.117 | 0.8 [0.6-1.1] | 0.221 | 0.9 [0.6-1.3] | 0.522 | - | - |
| Stroke (yes) | | 0.9 [0.6-1.2] | 0.371 | - | - | 1.4 [0.9-2.1] | 0.109 | 1.6 [0.8-2.9] | 0.163 |
| Vascular disease (Yes) | | **0.7 [0.5-0.9]** | **0.009** | 0.9 [0.6-1.3] | 0.550 | 0.7 [0.5-0.9] | 0.012 | 0.8 [0.5-1.3] | 0.420 |
| CHA2DS2VA-score | | 0.9 [0.8-0.9] | <0.001 | 1.0 [0.9-1.2] | 0.971 | 0.9 [0.8-0.99] | 0.020 | 0.9 [0.7-1.1] | 0.406 |
| ORBIT-score | Low | 1 |  | 1 |  | 1 |  |  |  |
|  | Medium and high | **0.5 [0.4-0.7]** | **<0.001** | 0.7 [0.5-0.9] | **0.022** | **0.5 [0.3-0.6]** | **<0.001** | 0.7 [0.5-1.1] | 0.104 |
| Admission haemoglobin, (g/L) | | **1.01 [1.00-1.01]** | **0.008** | 1.01 [0.99-1.01] | 0.493 | **1.01 [1.01-1.02]** | **<0.001** | 1.01[0.99-1.01] | 0.172 |
| Length of hospital stay (days) | | **-** | **-** | - | - | 0.99 [0.99-1.00] | 0.082 | 1.00 [0.99-1.01] | 0.890 |

AF, atrial fibrillation; CHF, congestive heart failure; *Including only variables eligible for multivariate analysis

Table S2: Specific group on discharge according to specific group on admission

| **At admission** | **At discharge** | | | | | | | | | |
| --- | --- | --- | --- | --- | --- | --- | --- | --- | --- | --- |
|  | Correct DOAC Dose | Correctly receiving warfarin | Correctly not receiving any OAC | Receiving DOAC at overdose | Receiving DOAC at under dose | Receiving AC despite CI | Lacking OAC | Receiving warfarin despite no CI to DOAC | Receiving DOAC with other error | Total |
| Correct DOAC Dose | **371 (95.1)** | 0 (0.0) | 0 (0.0) | 1 (0.3) | 8 (2.1) | 1 (0.3) | 8 (2.1) | 1 (0.3) | 0 (0.0) | 390 |
| Correctly receiving warfarin | 0 (0.0) | **23 (92.0)** | 1 (4.0) | 0 (0.0) | 0 (0.0) | 0 (0.0) | 1 (4.0) | 0 (0.0) | 0 (0.0) | 25 |
| Correctly not receiving any OAC | 3 (7.7) | 2 (5.1) | **32 (82.1)** | 0 (0.0) | 0 (0.0) | 1 (2.6) | 0 (0.0) | 0 (0.0) | 1 (2.6) | 39 |
| Receiving DOAC at overdose | 8 (42.1) | 0 (0.0) | 0 (0.0) | **9 (47.4)** | 1 (5.3) | 1 (5.3) | 0 (0.0) | 0 (0.0) | 0 (0.0) | 19 |
| Receiving DOAC at under dose | 15 (25.0) | 0 (0.0) | 0 (0.0) | 0 (0.0) | **42 (70.0)** | 0 (0.0) | 3 (5.0) | 0 (0.0) | 0 (0.0) | 60 |
| Receiving AC despite CI | 4 (9.1) | 5 (11.4) | 13 (29.5) | 0 (0.0) | 0 (0.0) | **18 (40.9)** | 4 (9.1) | 0 (0.0) | 0 (0.0) | 44 |
| Lacking OAC | 116 (50.0) | 16 (6.9) | 0 (0.0) | 2 (0.9) | 7 (3.0) | 4 (1.7) | **86 (37.1)** | 1 (6.7) | 0 (0.0) | 232 |
| Receiving warfarin despite no CI to DOAC | 14 (46.7) | 0 (0.0) | 0 (0.0) | 0 (0.0) | 1 (3.3) | 0 (0.0) | 2 (6.7) | **13 (43.3)** | 0 (0.0) | 30 |
| Receiving DOAC with other error | 0 (0.0) | 0 (0.0) | 0 (0.0) | 0 (0.0) | 0 (0.0) | 0 (0.0) | 0 (0.0) | 0 (0.0) | **3 (100)** | 3 |
| n | 531 | 46 | 46 | 12 | **59** | 25 | **106** | 15 | 4 | **844** |

Table S3. Changes of admission inappropriate stroke-prevention therapy during hospitalisation as assessed at discharge.

| Patient variables | | | | Overall  [n = 390, 100%] | Appropriateness status of anticoagulant therapy at discharge | | Between group P-value |
| --- | --- | --- | --- | --- | --- | --- | --- |
|  |  |  |  |  | Appropriate [n = 191, 49.0%] | Not appropriate [n = 199, 51.0%] |  |
| Gender, n (%) | Male | | | 238 (61.0) | 129 (54.2) | 109 (45.8) | 0.010 |
|  | Female | | | 152 (39.0) | 62 (40.8) | 90 (59.2) |  |
| Age (yr.), mean [SD] | | | | 77.4 (10.9) | 75.3 (11.2) | 79.5 (11.2) | <0.001 |
| Cause of admission | Stroke | | | 49 (12.6) | 38 (77.6) | 11 (22.4) | < 0.001 |
|  | Atrial fibrillation | | | 36 (9.2) | 22 (61.1) | 14 (38.9) |  |
|  | Surgery unrelated to stroke | | | 97 (24.9) | 42 (43.3) | 55 (56.7) |  |
|  | Other medical admissions | | | 208 (53.3) | 89 (42.8) | 119 (57.2) |  |
| Admission ward | Stroke | | | 51 (13.1) | 42 (82.4) | 9 (17.6) | < 0.001 |
|  | Cardiology | | | 63 (16.2) | 45 (71.4) | 18 (28.6) |  |
|  | Surgical | | | 96 (24.6) | 44 (45.8) | 52 (54.2) |  |
|  | General medicine | | | 135 (34.6) | 48 (35.6) | 87 (64.4) |  |
|  | Geriatric | | | 18 (4.6) | 5 (27.8) | 13 (72.2) |  |
|  | Emergency | | | 6 (1.5) | 2 (33.3) | 4 (66.7) |  |
|  | Oncology | | | 11 (2.8) | 2 (18.2) | 9 (81.8) |  |
|  | Others | | | 10 (2.6) | 3 (30.0) | 7 (70.0) |  |
| CHA_2_DS_2_VA-score, mean (SD) | | | | 4.0 (1.5) | 3.9 (1.5) | 4.0 (1.5) | 0.638 |
| ORBIT score, mean (SD) | | | | 2.5 (1.5) | 2.3 (1.5) | 2.7 (1.4) | 0.004 |
| ORBIT score —categorical, n (%) | | Low (0-2) | | 187 (47.9) | 106 (56.7) | 81 (43.3) | <0.014 |
|  |  | Medium (3) | | 93 (23.9) | 39 (41.9) | 54 (58.1) |  |
|  |  | High (≥4) | | 110 (28.2) | 46 (41.8) | 64 (58.2) |  |
| CHF, n (%) | | | | 125 (32.1) | 66 (52.8) | 59 (47.2) | 0.299 |
| Hypertension, n (%) | | | | 263 (67.4) | 129 (49.0) | 134 (51.0) | 0.966 |
| Diabetes, n (%) | | | | 112 (28.7) | 54 (48.2) | 58 (51.8) | 0.849 |
| Stroke, n (%) | | | | 86 (22.1) | 54 (62.8) | 32 (37.2) | 0.004 |
| Vascular disease, n (%) | | | | 260 (66.7) | 123 (47.3) | 137 (52.7) | 0.352 |
| Bleeding history, n (%) | | | | 27 (6.9) | 18 (66.7) | 9 (33.3) | 0.057 |
| CKD, n (%) | | | | 64 (16.4) | 27 (42.2) | 37 (57.8) | 0.235 |
| Admission haemoglobin, (g/L), mean (SD) | | | | 126.4 (22.9) | 129.9 (22.8) | 123.0 (22.5) | 0.003 |
| Admission platelets (×10^9^/L), mean (SD) | | | | 240.7 (99.5) | 241.0 (100.8) | 240.4 (98.4) | 0.954 |
| Admission eGFR (mL/min), mean (SD) | | | | 64.2 (23.0) | 65.2 (23.5) | 63.2 (22.6) | 0.399 |
| Admission creatinine (µmol/L), mean (SD) | | | | 105.3 (81.4) | 105.7 (75.6) | 104.9 (86.8) | 0.924 |
| Presence of clinical outcomes | | | Yes | 57 (14.6) | 37 (64.9) | 20 (35.1) | 0.009 |
|  |  |  | No | 333 (85.4) | 154 (46.2) | 179 (53.8) |  |
| Specific clinical outcomes | | | Bleeding events | 32 (56.1) | 23 (71.9) | 9 (28.1) | 0.007 |
|  |  |  | Thromboembolic events | 26 (45.6) | 15 (57.7) | 11 (42.3) | 0.357 |
| Admission anticoagulant therapy, n (%) | | | Nil OAC | 232 (59.5) | 132 (56.9) | 100 (43.1) | < 0.001 |
|  |  |  | Apixaban | 57 (14.6) | 26 (45.6) | 31 (54.4) |  |
|  |  |  | Rivaroxaban | 63 (16.2) | 16 (25.4) | 47 (74.6) |  |
|  |  |  | Dabigatran | 6 (1.5) | 1 (16.7) | 5 (86.3) |  |
|  |  |  | Warfarin | 32 (8.3) | 16 (50.0) | 16 (50.0) |  |
| Re-hospitalisation within 3 months | | | Yes | 138 (35.4) | 70 (50.7) | 68 (49.3) | 0.609 |
|  |  |  | No | 252 (64.6) | 121 (48.0) | 131 (52.0) |  |
| Cause of re-hospitalisation | | | Bleeding | 7 (5.1) | 3 (42.9) | 4 (57.1) | 0.801 |
|  |  |  | Thromboembolic events | 9 (6.5) | 1 (11.1) | 8 (88.9) | 0.043 |
